# Assessment of the Diagnostic Performance of MUAC in Malnutrition Screening and Its Correlation with Other Anthropometric Indicators in Healthy Children and Adolescents

**DOI:** 10.3390/children11121535

**Published:** 2024-12-18

**Authors:** Hatice Esra Durukan, Burçe Emine Dörtkardeşler, Merve Tosyalı, Şule Gökçe, Nuri Zafer Kurugöl, Feyza Koç

**Affiliations:** 1 Division of General Pediatrics, Department of Pediatrics, Faculty of Medicine, Children’s Hospital, Ege University, 35100 Izmir, Turkey; h.esra00@gmail.com (H.E.D.); sule.gokce@ege.edu.tr (Ş.G.); zafer.kurugol@ege.edu.tr (N.Z.K.); 2 Division of Social Pediatrics, Department of Pediatrics, Faculty of Medicine, Children’s Hospital, Ege University, 35100 Izmir, Turkey; merve.tosyali@ege.edu.tr (M.T.); feyza.koc@ege.edu.tr (F.K.)

**Keywords:** body mass index, malnutrition, mid-upper arm circumference, obesity, undernutrition

## Abstract

Background/Objectives: This study aimed to evaluate the correlation of mid-upper arm circumference (MUAC) z-scores with body mass index (BMI) and weight-for-height (WFH) z-scores to determine its reliability in identifying malnutrition and its potential in clinical practice in healthy children and adolescents. Methods: Our study included 906 healthy children and adolescents aged between 2 months and 18 years who were admitted to University Hospital’s General Pediatrics Clinic and attended 12 primary schools in 6 additional Izmir provinces. Anthropometric measurements (weight, length/standing height, MUAC, BMI, WFH) were performed. The relationship between MUAC z-scores, BMI, and WFH z-scores of cases with malnutrition were evaluated. Results: According to the WHO BMI z-score classification, 6 (0.7%) of the children were defined as having severe undernutrition, 43 (4.7%) as moderate undernutrition, 146 (16.1%) as mild undernutrition, 486 (53.6%) as normal, 142 (15.7%) as overweight, and 83 (9.2%) as obese. At any age over two years, fair agreement was observed between MUAC z-scores and WHO BMI z-scores in defining malnutrition alone compared to other growth measures (weighted kappa = 0.371). Under two years of age, the correlation between MUAC z-scores and BMI z-scores showed moderate agreement in detecting overweight and obesity (weighted kappa = 0.479), and between MUAC and WHO WFH z-scores showed moderate agreement (kappa = 0.252). Conclusions: The study found a moderate and fair connection between MUAC z-scores and other criteria. However, further MUAC z-score screening and diagnostic power testing in larger pediatric populations are needed to validate its use alongside other key anthropometric indicators in malnutrition diagnosis. MUAC measurement should be popularized in routine pediatric outpatient clinics to detect malnutrition quickly.

## 1. Introduction

Malnutrition, resulting from an unbalanced intake of proteins, calories, fats, and other essential nutrients required for growth and development, affects all body systems and has profound cellular-level consequences. Global challenges such as poverty, scarcity, and war severely hinder food distribution and access, making food utilization difficult and leading to primary malnutrition. Malnutrition remains a significant global health issue, accounting for 45% of deaths among children under five years of age. Approximately 45 million children worldwide under the age of five are undernourished, with around 20 million facing severe undernutrition [1].

Primary malnutrition is defined as a food supply problem caused by socioeconomic factors, how the country is governed, and sometimes environmental factors such as natural disasters and the inability to access food [2]. Malnutrition in the presence of an underlying disease, such as increased energy requirements, impaired digestion, or absorption of nutrients, is defined as secondary malnutrition [3]. It has been reported that approximately one-third of children admitted to hospitals in developed countries are malnourished. Malnutrition prolongs hospitalization if left untreated and causes significant clinical symptoms such as susceptibility to infections and delayed recovery [4].

On the spectrum of obesity, defined as a severe issue caused by the deposition of excessive fat in the body, it has been reported that 37 million children under the age of five are overweight. Additionally, over 390 million children and adolescents aged 5–19 years are overweight, including 160 million living with obesity [5]. In addition, it has also been reported that overweight children under the age of five globally face various morbidity risks each year [6].

The Non-Communicable Disease Risk Factor Collaboration (NCD-RisC) conducted a pooled analysis of 2181 studies across 200 countries from 1985 to 2019, examining trends in height and body mass index (BMI) [7]. The study highlighted that in some countries, children who started at age five with height and BMI appropriate for their age failed to achieve adequate growth over time or gained excessive weight relative to their height, leading to poorer health outcomes. These findings underscore the increasing prominence of chronic undernutrition, resulting in stunted growth or obesity over the years.

Another study by the NCD-RisC group analyzed data from 3663 population-representative studies conducted between 1990 and 2022 to detect trends in underweight and obesity [8]. The findings revealed that the prevalence of both obesity and thinness in children and adolescents has been increasing in most countries. By 2022, obesity prevalence exceeded thinness prevalence in 133 countries among girls and 125 countries among boys. The study highlighted that rising rates of obesity were the primary drivers of the dual burden of malnutrition [8]. These findings emphasize the critical need for comprehensive and targeted strategies to address both undernutrition and overnutrition, underscoring the urgency of tackling the global dual burden of malnutrition.

Malnutrition, encompassing both undernutrition and overnutrition, not only results in mortality but also imposes significant economic, social, and medical burdens on countries and societies. Unfortunately, some of these negative consequences are permanent [9]. For these reasons, malnutrition, with a spectrum of both undernutrition and overweight/obesity, remains a severe public health problem worldwide [10]. To maintain preventive health services that continue regularly and with high quality; and to create a healthy society, every child must be examined in detail with regard to malnutrition when they present to a health institution for any reason, from infancy to adulthood [1]. Anthropometric measurements and growth criteria are essential for detecting undernutrition and overweight/obesity. These measurements include weight, length or standing height, BMI, growth percentile curves, weight-for-height (WFH), weight-for-age (WFA), standard deviation percentiles, z-scores, mid-upper arm circumference (MUAC), and skinfold thickness (especially triceps and subscapular measurements). Growth criteria and rate calculations are typically conducted using each country’s specific growth curves.

The origins of MUAC measurement date back to the late 1950s when it was first utilized in Haiti by a tropical medicine specialist and his team from the Caribbean Food and Nutrition Institute. A significant milestone in its development and application occurred in 1969, establishing MUAC as an essential tool in nutritional assessment [11]. MUAC is widely used as an anthropometric measure because it provides a simple and quick method to identify children at high risk of malnutrition without requiring adjustments for age or sex. The World Health Organization (WHO) highlights MUAC as a critical diagnostic tool for detecting malnutrition in children aged 6–59 months, noting its superior ability to predict morbidity and mortality compared to BMI and weight-for-height z-scores [9,12]. With their affordability and practicality, MUAC measuring strips have become an ideal screening tool for diagnosing and monitoring malnutrition in the pediatric population [13]. While internationally agreed-upon cut-offs for children older than five years are currently lacking, an investigation by Rahman et al. has partially provided clinicians with data to calculate MUAC z-scores, showcasing the potential of MUAC to evaluate the nutritional status of children and adolescents across all age groups [9].

In our study, we aim to assess the diagnostic accuracy and reliability of MUAC in identifying undernutrition and overweight/obesity among healthy children and adolescents aged 2 months to 18 years. Additionally, we aim to evaluate the correlation of MUAC z-scores with BMI and weight-for-height z-scores to determine its potential utility in clinical practice. This investigation seeks to contribute to the growing body of evidence supporting MUAC as a practical, cost-effective tool for monitoring nutritional status in pediatric populations.

## 2. Materials and Methods

Our study was planned as an analytical cross-sectional study to evaluate the correlation between MUAC z-scores and z-scores of other growth measures (WFH and BMI) in children and adolescents aged two months to 18 years. The study was approved by the local ethics committee of Ege University (22-6.1T/45). The study was conducted in accordance with the guidelines of the Declaration of Helsinki. Verbal and written consent was obtained from both the children and adolescents who participated in the study and their legal guardians.

### 2.1. Data Collection

Between 1 July 2022 and 1 January 2023, anthropometric measurements—including length/standing height, weight, and mid-upper arm circumference (MUAC)—were collected from children and adolescents aged 2 months to 18 years in Izmir, Turkey. Children and adolescents aged 6 to 18 years were enrolled from schools located in six districts of Izmir, with three districts representing low socioeconomic conditions and three districts representing high socioeconomic conditions, chosen to capture a range of sociodemographic characteristics. Children aged 2 months to 6 years were selected from patients presenting to the General Pediatrics Outpatient Clinic at Ege University Hospital, a tertiary care center. All measurements were conducted by the same researcher to ensure consistency, and data on anthropometric measurements and sociodemographic characteristics were recorded using standardized case report forms.

A power analysis was conducted prior to initiation. Under the null hypothesis (H_0_), the correlation coefficient between MUAC and WFH and BMI z-scores was assumed to be 0.3 (moderate effect size), while under the alternative hypothesis (H_1_), it was hypothesized to be 0.4. Based on this analysis, the minimum required sample size for a two-tailed hypothesis test with a significance level of 0.05 and 80% power was determined to be 605 participants. To eliminate the confounding effects of sex and age factors, we planned to include an equal number of participants across all age and sex groups, resulting in a target sample size of 864 participants. With an additional 10% margin for potential errors, the final sample size was planned to be 950 participants.

### 2.2. Exclusion Criteria

Children who declined to participate, whose consent could not be obtained from themselves or their families, or who had chronic diseases were excluded from the study. Additionally, children with incomplete measurements or missing sociodemographic data were also excluded. A total of 44 participants were excluded from the study.

### 2.3. Anthropometric Measurement Methods and Interpretation of Data

In children up to two years of age, body weight was measured using an infant scale sensitive to a 10 g change. The measurement was taken with the infant lying down or, for those who could sit without support, in a seated position. In children older than two years and adolescents, body weight was measured using a standard scale sensitive to a 100 g change. The same scale was used for all children over two years to ensure consistency. Calibration of all measuring instruments was performed at regular intervals to maintain accuracy [14,15,16].

The length of children under 2 years of age was measured with the child in a supine position, using a headboard and footboard to determine the distance from the top of the head to the soles of the feet. For children older than two years, standing height was measured in a standing position using a stadiometer. During standing measurements, the child was positioned with their back straight, heels together, and feet flat on the floor, ensuring the head was aligned in the Frankfurt plane (where the lower margin of the orbit is in line with the upper margin of the external auditory meatus). This positioning ensured accurate and standardized standing height measurements across all participants [14,15,16].

**Figure 1:**
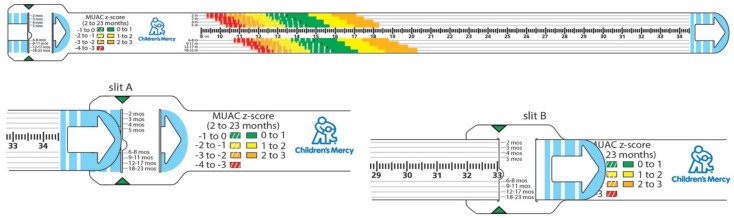
Prototype MUAC Z-score tape [13].

To measure MUAC, a non-stretchable insertion tape graduated in millimeters was used. The measurement process was conducted on the child’s non-dominant arm, which was determined based on the statements of the children and adolescents and/or their families and further confirmed through observation. For children under the age of one, where the dominant arm could not be clearly identified, the left arm was used. First, the child’s arm was bent at a 90-degree angle at the elbow to identify the midpoint. The arm was held parallel to the side of the body, and the length of the upper arm was measured from the shoulder tip (acromion) to the elbow tip (olecranon process). The same measuring tape was used as a ruler to determine this length, and the midpoint between these anatomical landmarks was marked. Next, with the participant’s arm hanging loosely by their side, the circumference of the upper arm was measured at the marked midpoint. The tape was placed snugly around the arm, ensuring it was neither too tight nor too loose. The measurement was recorded to the nearest millimeter [17]. For interpreting MUAC measurements, z-score-based MUAC bands designed for children and adolescents aged two months to 18 years were used, with measurements categorized by the color-coded sections on the band (Table 1 and Figure 1).

Growth criterion parameters obtained from anthropometric measurements according to age and sex were identified as follows [18]:

In the undernutrition part of malnutrition, BMI z-score:

- below −3 was defined as “severe undernutrition”,
- between −3 and −2 as “moderate undernutrition”,
- between −2 and −1 as “mild undernutrition”.

In the undernutrition part of malnutrition, WFH percentile and z-scores according to WHO data in children under five years of age [18,19]:

- between 90–110% was considered “normal”,
- 80–90% were defined as “acute mild malnutrition”,
- 70–80% as “acute moderate malnutrition”,
- below 70% as “acute severe malnutrition”,
- between −2 and −1 as “acute mild malnutrition”,
- between −3 and −2 as “acute moderate malnutrition”,
- below −3 z-score was defined as “acute severe malnutrition”.

For the overweight/obesity part of malnutrition, children over the age of two with a BMI [5,20];

- 85–95th percentile is defined as “overweight”,
- above the 95th percentile are defined as “obese”.

Class I obesity is defined as having a BMI ≥ 95th percentile but less than 120% of the 95th percentile, or a BMI ≥ 30 but less than 35 (whichever is lower).

Class II obesity is defined as having a BMI ≥ 120% but less than 140% of the 95th percentile, or a BMI ≥ 35 but less than 40 (whichever is lower).

- 120% of the 95th percentile corresponds approximately to the 98th percentile or a BMI z-score ≥ 2).

Class III obesity is defined as having a BMI ≥ 140% of the 95th percentile, or a BMI ≥ 40 (whichever is lower). 

### 2.4. Statistical Analysis

Statistical analyses were performed with IBM^®^ SPSS^®^ 26 (SPSS Inc., Chicago, IL, USA) software. The conformity of numerical variables to normal distribution was examined by the Kolmogorov-Smirnov test (n ≥ 50). Numerical variables were presented as mean ± standard deviation or median (min-max), depending on their distribution. Categorical data were summarized using numbers and percentages. In statistical analyses, the chi-square test, *t*-test, paired sample *t*-test, Fisher exact test, or Mann-Whitney U test were used according to the determined data. Pearson’s chi-square analysis or Fisher’s exact chi-square analysis was used to analyze categorical data. The effect of independent variables on the dependent variable was shown by chi-square analysis, and logistic regression models explained the impact of significant independent variables on the dependent variable. In all cases, a *p*-value < 0.05 was considered significant. The correlation between variables and coefficients of concordance was analyzed using Kappa and weighted Kappa analysis. Weighted Kappa analysis was evaluated for ordinal data. Kappa analysis evaluation was performed according to the scale (Table 2) [21]. Kappa values between 0.61–0.80 and above were considered significant correlations.

## 3. Results

### 3.1. Demographic Data

In this study, 906 children and adolescents were involved in the General Pediatrics Outpatient Clinic of a tertiary University’s Children’s Hospital and attended 12 different primary schools in six other districts of Izmir province. Of the included children, 456 (50.3%) were boys and 450 (49.7%) were girls. The median age was 9.27 years (min–max: 0.19–17.97 years). There were 100 (11%) children under two years of age and 806 (89%) children over two years of age. Of the families, 794 (87.6%) were nuclear families and 112 (12.4%) were extended families. There were 489 (54%) children whose mothers had a high school education or above. 819 (90.4%) of the children lived in the city center. Family income level was grouped according to the hunger and poverty line at the time of measurement; 140 (15.5%) were low-income, and 674 (74.4%) were middle-income. All demographic data are shown in Table 3.

### 3.2. Relationship Between Demographic Data and Undernutrition and Overweight/Obesity

A significant difference was found between undernutrition and overweight/obesity with respect to sex in the classification made using MUAC z-scores when analyzing the relationship between demographic data and undernutrition/obesity in all children and adolescents included in the study (*p* < 0.05). In the classification made using WHO BMI z-scores, a significant difference was also found between sex, undernutrition, and obesity (*p* < 0.05). While the obesity rate was significantly higher in boys, undernutrition was more common in girls.

In the relationship between the demographic data of the children included in the study and undernutrition and overweight/obesity, a significant difference was found between sex and undernutrition and overweight/obesity in children aged two years and older (according to the classification based on BMI z-scores by the Center for Disease Control and Prevention (CDC)). In contrast, there was no significant difference between sex and undernutrition and overweight/obesity in children under two years of age (in classification using BMI and BGA z-scores according to WHO) (*p* < 0.05, *p* > 0.05, respectively). A significant relationship was found between female sex and undernutrition and male sex and overweight/obesity in the classification based on MUAC z-scores, WHO BMI z-scores, and CDC BMI z-scores (*p* < 0.05).

In all children included in the study, no significant correlation was found between undernutrition and overweight/obesity classification based on MUAC z-scores and WHO BMI z-scores and duration of pregnancy, mother’s number of births, family type, maternal and paternal education, income level, and place of residence (*p* > 0.05).

### 3.3. Nutritional Status of the Subjects

In the study, 486 (53.6%) of the children were found to be in the normal range, 142 (15.7%) overweight, and 83 (9.2%) obese, according to the WHO BMI z-score growth standards. Additionally, 6 (0.7%), 43 (4.7%), and 146 (16.1%) children were classified as severely undernourished, moderately undernourished, and mildly undernourished, respectively. Stunting was observed in 12% of children under two years of age and 8.6% of children aged two years and older. In the height-for-age evaluation of the patients, 5 (0.6%) had severe chronic undernutrition, 11 (1.2%) had moderate chronic undernutrition, 65 (7.2%) had mild chronic undernutrition, and 825 (91.1%) were normal. According to CDC BMI z-scores of children aged two years and older, 424 (52.6%) of the children were diagnosed as normal, 207 (25.6%) as undernourished, 152 (18.9%) as overweight, and 23 (2.9%) as obese. The classification of MUAC z-scores at all ages and BMI z-scores according to WHO growth standards is summarized in Table 4.

### 3.4. Correlation Analyses of Mid-Upper Arm Circumference and Other Growth Measures

In detecting the presence of undernutrition, the sensitivity of MUAC z-scores across all ages was compared to WHO BMI z-scores, with a sensitivity of 69.2% and a specificity of 83.7%. For children under 2 years of age, the sensitivity of MUAC z-scores in detecting undernutrition was 82.4% and specificity 73.5% compared to WHO BMI z-scores. For children two years and older, the sensitivity of MUAC z-scores in detecting undernutrition was 67.2% and specificity 90.3% compared to CDC BMI z-scores; the sensitivity was 60.9% and specificity 85.1%.

Table 5 shows the result of correlation analyses of the malnutrition classification between MUAC z-scores and WHO BMI z-scores, which were found to have a weighted kappa of 0.371 for all ages. In the classification of undernutrition, normal, and overweight/obesity based on the correlation between MUAC and WHO BMI z-scores, the weighted kappa value was found to be 0.398, indicating fair agreement for all ages. 

In the undernutrition present/no field, there was a moderate agreement among those older than two years (weighted kappa = 0.493). In the correlation analysis between MUAC and WHO BMI z-scores, the weighted kappa was 0.419, which is defined as a moderate agreement in children under two years of age. There was a fair agreement on correlation analysis between MUAC and WHO BMI z-scores older than two years (see Table 6).

## 4. Discussion

Malnutrition is a nutritional disorder resulting from failure to provide sufficient/balanced nutrition for growth and development. Malnutrition causes 45% of deaths in children under five years of age worldwide [10]. It is reported that one out of every three people in the world are affected by subgroups of undernutrition such as wasting, stunting, micronutrient deficiency, or obesity. In our study, 486 (53.6%) of the children were found to be normal, 142 (15.7%) overweight, and 83 (9.2%) obese according to the World Health Organization BMI z-score growth standards. In addition, 6 (0.7%), 43 (4.7%), and 146 (16.1%) children were severely undernourished, moderately undernourished, and mildly undernourished, respectively.

According to the analysis data published by the WHO, United Nations Children’s Fund (UNICEF) and World Bank Group in 2021, 5.7% of children under five years of age were overweight, 6.7% were undernourished, and 22% were stunted [6]. In the Turkish Demographic and Health Surveys, it was found that 6% of children under five years of age were stunted, 2% were undernutrition, and 8% were overweight, using WHO z-score growth standards [22]. In our study, prevalence of stunting was 12% in children under two years of age and 8.6% in children aged two years and older. According to the WHO’s WFH criteria percentages, the undernutrition rate was 13%, the overweight rate was 15% in children under two years of age, and the undernutrition rate was 17% in children over two years of age. In the height-for-age evaluation of our patients, 5 (0.6%) had severe chronic undernutrition, 11 (1.2%) had moderate chronic undernutrition, 65 (7.2%) had mild chronic undernutrition, and 825 (91.1%) were normal. In the height-for-age classification, undernutrition and overweight rates were higher in our study than in the global and Turkish data, whereas stunting rates were lower than in the international data. It is thought that this may be because the nutritional characteristics of the participants below the age of two years and the effect of birth length were not evaluated. Additionally, the fact that most of our participants were patients under the age of five years was also thought to affect this rate.

Our study evaluated the correlation of MUAC measurement, which is accepted as a screening tool for malnutrition in healthy children and adolescents, with WHO and CDC growth criteria. In the correlation of MUAC z-scores with WHO WFH z-scores in children under two years of age, the coefficient of concordance was statistically interpreted as fair agreement. The agreement was moderate in the correlation between the MUAC and WHO BMI z-scores. Moderate agreement in kappa values typically indicates a fair to good agreement that implies some consistency or pattern in the relationship between the studied variables. However, there may still be some variability or disagreement between them. This indicates that the strength of agreement between MUAC and WHO BMI z-scores varies slightly between age groups, with a slightly stronger agreement observed in younger children compared to older subjects. However, measuring MUAC under two years of age may be technically inaccurate (due to the mobility of the child, etc.). Therefore, it was thought that MUAC measurement may be more appropriate for screening purposes in children over two years of age.

In our study, when undernutrition and overweight/obesity were defined without subgroups, a low level of agreement was obtained between MUAC z-scores and WHO BMI z-scores at all ages, and no significant correlation was found between MUAC and growth measures at all ages and above two years of age in defining the presence of obesity alone. The correlation analysis of MUAC and CDC BMI z-scores suggests that MUAC measurement is less valuable in detecting overweight/obesity at two years and older. In conclusion, as mentioned above, MUAC measurement showed low detection of undernutrition under two years of age and obesity over two years of age. In addition, it was concluded that it would be more appropriate to use BMI to screen for overweight/obesity in children aged two years and older. Currently, the American Academy of Pediatrics (AAP) and CDC recommend the use of WHO growth curves until the age of two years and CDC growth curves after the age of two years (2–20 years) [23,24].

Briend et al. reported that MUAC measurement may be a better marker than WFH in detecting children with severe undernutrition, and MUAC measurement can be used alone [2,13]. In another study conducted by Mramba et al. with school children and adolescents, it was reported that MUAC measurement was as effective as BMI measurement in assessing malnutrition and mortality risk. This risk could be quickly evaluated with MUAC [25]. Studies have shown that the MUAC z-score band is a feasible, practical, and low-cost alternative method for malnutrition screening [13,26,27]. However, in a study published in 2017, Bouma argues that MUAC may be moderately associated with both fat and lean mass independent of height in healthy infants, but may not capture the diagnosis of malnutrition in stunted children who gain weight [28]. Similarly, our study found that the correlation of MUAC measurement with WHO and CDC growth criterion z-scores was partially sufficient. The main limitation of our study is that it was conducted in a limited area and on a small number of children in Izmir, Turkey. Therefore, although MUAC measurement is recommended for identifying malnutrition and follow-up of nutrition programs, large-scale studies on using MUAC measurement alone and correlation analysis are needed.

There is growing interest in determining whether body composition parameters are more effective than traditional anthropometric measurements in identifying malnutrition in pediatric patients [29]. Body composition can be assessed in complex pediatric cases using various techniques, with bioelectrical impedance analysis (BIA) emerging as a promising alternative for evaluating fat-free mass (FFM). BIA has proven to be a reliable and precise method for assessing FFM, fat mass (FM), and percentage body fat (PBF), particularly in pediatric populations [30]. Its rapid and non-invasive nature further enhances its clinical applicability, and the bioelectrical impedance phase angle is recognized as a valuable marker for nutritional assessment in children [31]. In conditions where height and weight measurements may not be reliable, such as chronic kidney disease, cirrhosis, or spastic cerebral palsy, BIA is often preferred for its ability to detect malnutrition earlier through direct FFM measurements [32,33,34]. This early detection capability is critical for timely intervention and improved clinical outcomes. Including BIA measurements in our study could have strengthened our findings by providing additional insight into the role of mid-upper arm circumference (MUAC) in early malnutrition detection. However, due to technical limitations, BIA measurements could not be obtained. Future research incorporating body composition assessments, including BIA, is planned to further explore this important area.

## 5. Conclusions

Malnutrition (undernutrition and overweight/obesity) continues to be a public health problem all over the world, including in our country. Although the correlation of MUAC z-scores determined in our study with other criteria was moderate to low, it can be used in daily practice due to its speed, simplicity, and ease of use. However, it was concluded that it would be more appropriate to evaluate MUAC with the z-scores used by the WHO and CDC to diagnose malnutrition. Large-scale studies are needed to determine the effectiveness of MUAC measurement in identifying children and adolescents at nutritional risk and the correlation between the MUAC z-score and the z-scores for conventional indicators (i.e., WFH and BMI).

## Figures and Tables

**Table 1:** Z-score classification according to MUAC bands.

Reference Table for z-Score Ranges on Tape		
Color/Pattern Key	MUAC z-Score Range	Risk Classification
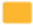 Solid Orange	2 to 3	Moderate overweight
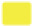 Solid Yellow	1 to 2	Mild overweight
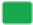 Solid Green	0 to 1	Normal
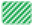 Hashed Green	−1 to 0	Normal
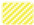 Hashed Yellow	−2 to −1	Mild Undernutrition
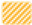 Hashed Orange	−3 to −2	Moderate Undernutrition
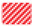 Hashed Red	−4 to −3	Severe Undernutrition

MUAC: Middle-Upper Arm Circumference.

**Table 2:** Kappa analysis evaluation scale [21].

Kappa Value	Criteria
<0	No agreement
0.00 to 0.20	Slight agreement
0.21 to 0.40	Fair agreement
0.41 to 0.60	Moderate agreement
0.61 to 0.80	Substantial agreement
0.81 to 1.00	Almost perfect agreement

**Table 3:** The general characteristics of the children and adolescents included in the study (*n* = 906).

		n (%)
Sex	Male	456 (50.3)
	Female	450 (49.7)
Age (year) *		9.17 ± 5.21
Week of birth *	Preterm	109 (12)
	Term	787 (86.9)
	Postterm	10 (1.1)
Birth weight (gr) *		3265.80 ± 582.96
Birth length (cm) *		49.62 ± 2.20
Duration of exclusive breastfeeding (months)		5.23 ± 1.53
Total duration of breastfeeding (months)		14.73 ± 9.24
Measurement location	Hospital	293 (32.3)
	School	613 (67.7)
Weight (kg) *		35.92 ± 21.36
Length/standing height (cm) *		133.01 ± 31.71
Ranking among siblings	First	470 (51.9)
	Second	333 (36.8)
	Third and later	100 (11)
Family structure	Nuclear	794 (87.6)
	Extended	112 (12.4)
Age of mothers (year) *		37.49 ± 6.76
Age of fathers (year) *		40.82 ± 7.05
The education level of mothers	Below high school	417 (46)
	High school and above	489 (54)
The education level of fathers	Below high school	362 (40)
	High school and above	544 (60)
Place of residence	Village/town	87 (9.6)
	City centre	819 (90.4)
Family economic situation	Low-income	140 (15.5)
	Middle-income	674 (74.4)
	High-income	92 (10.2)

* Mean ± SD.

**Table 4:** Undernutrition and overweight rates according to MUAC band and WHO at all ages.

**Classification for undernutrition**	**MUAC z-score** **n (%)**	**WHO BMI z-score** **n (%)**
Mild undernutrition	178 (19.6)	146 (16.1)
Moderate undernutrition	62 (6.8)	43 (4.7)
Severe undernutrition	11 (1.2)	6 (0.7)
**Classification for overweight**	**MUAC z-score** **n (%)**	**WHO BMI z-score** **n (%)**
Normal	586 (64.7)	486 (53.6)
Overweight	64 (7.1)	142 (15.7)
Obese	5 (0.6)	83 (9.2)

MUAC: Middle-upper Arm Circumference; WHO: World Health Organization; BMI: Body Mass Index.

**Table 5:** Correlations and numbers of cases according to MUAC and WHO BMI z-scores.

	MUAC Z-Score	Severe Undernutrition	Moderate Undernutrition	Mild Undernutrition	Normal	Overweight	Obese	Total (n)
WHO BMI Z-Score								
Severe undernutrition		3	2	1	0	0	0	6
Moderate undernutrition		5	20	16	2	0	0	43
Mild undernutrition		3	28	57	58	0	0	146
Normal		0	12	104	356	13	1	486
Overweight		0	0	0	126	16	0	142
Obese		0	0	0	44	35	4	83
Total (n)		11	62	178	586	64	5	906

Weighted kappa = 0.371. MUAC mid-upper arm circumference; WHO World Health Organization; BMI body mass index.

**Table 6:** Correlation analysis results of MUAC z-score and WHO BMI z-score.

		Kappa Level of <2 Years	Kappa Level of >2 Years
MUAC z-score and WHO BMI z-score	Whole study group	0.419	0.367
	Undernutrition/Normal/Obese	0.423	0.397
	Undernutrition Present/No	0.387	0.493
	Overweight/Obesity Present/No	0.479	0.275

kappa: weighted kappa. MUAC mid-upper arm circumference; WHO World Health Organization; BMI body mass index.

## Data Availability

The data presented in this study are available on request from the corresponding author.
